# Construction of ceRNA network and identification of hub genes in aniridia-associated keratopathy using bioinformatics analysis

**DOI:** 10.3389/fgene.2022.997581

**Published:** 2022-09-23

**Authors:** Jiawen Wu, Daowei Zhang, Jihong Wu, Shenghai Zhang

**Affiliations:** ^1^ Eye Institute, Eye and ENT Hospital, College of Medicine, Fudan University, Shanghai, China; ^2^ Shanghai Key Laboratory of Visual Impairment and Restoration, Science and Technology Commission of Shanghai Municipality, Shanghai, China; ^3^ State Key Laboratory of Medical Neurobiology, Institutes of Brain Science and Collaborative Innovation Center for Brain Science, Shanghai, China; ^4^ Key Laboratory of Myopia, Ministry of Health, Shanghai, China

**Keywords:** AAK, ocular surface, ceRNA network, immune microenvironment, biomarkers, WGCNA, IL-17 signaling pathway

## Abstract

Aniridia-associated keratopathy (AAK) is characteristic at ocular surface of aniridia caused by haploinsufficiency of *PAX6*. Competing endogenous RNA (ceRNA) has been reported to play an important role in various diseases, whereas its function on AAK is unclear. The microarray data of 20 AAK patients and 20 healthy people were downloaded from the Gene Expression Omnibus (GEO) database. Differentially expressed lncRNAs, miRNAs, and mRNAs were analyzed using “limma” packages and weighted gene co-expression network analysis (WGCNA). A ceRNA network was constructed by Cytoscape 3.9.1, and miR-224-5p, miR-30a-5p, and miR-204-5p were at the center of the network. CIBERSORTx algorithm and ssGSEA analyses revealed that AAK was associated with immune cell infiltration, showing that activated Mast cells increased while resting Mast cells decreased and NK cells decreased in AAK. Type II INF Response, CCR, parainflammation, T cell co-stimulation, and APC co-stimulation of AAK patients differed from healthy individuals. Additionally, the ROC curve of five genes, *MITF*(AUC = 0.988)*, RHOB*(AUC = 0.973)*, JUN*(AUC = 0.953)*, PLAUR* (AUC = 0.925)*,* and *ARG2* (AUC = 0.915) with high confidence in predicting AAK were identified. Gene set enrichment analysis (GSEA) analysis of hub genes enriched in the IL-17 signaling pathway.

## Introduction

Aniridia is a rare hereditary disorder caused by the haploinsufficiency of *PAX6* that may affect most structures of the eyes (Lim et al., 2017). Among these progressive pathologies, the ocular surface can suffer severe impairments during eye development by AAK (2). According to the recommendations of relevant researchers, AAK subtypes could be considered separate diseases, thereby facilitating treatment decisions and patient stratification for future clinical studies and trials (Käsmann-Kellner and Seitz, 2014).

Phenotypes of epithelial, neural, immune, and limbal stem cell status have recently been extensively studied in phenotypic AAK (Lagali et al., 2018). A previous study showed that more than 400 unique mutations in the PAX6 gene may lead to a series of clinical phenotypes (Lim et al., 2012). As most studies considered mutations in *PAX6* as a homogeneous group, other genes may interact with it, or the variation of downstream genes of *PAX6* may cause specific phenotypes (Lee and Colby, 2013). Hence, these heterogeneous gene alterations require more attention.

Microarray data analysis is used to analyze the mechanism of disease progression to improve diagnosis and treatment. Salmena et al. (2011) proposed the ceRNA hypothesis that various types of RNAs can control gene expression at the post-transcriptional level , meaning lncRNAs competitively bind to miRNAs, thereby up-regulating the translation of the corresponding mRNAs (Zhang et al., 2019). These ceRNA networks may reveal novel mechanisms promoting transcriptional regulatory networks for disease development and have been studied in many ophthalmic diseases (Salmena et al., 2011; Ye et al., 2017; Wang et al., 2021). However, the expression patterns of specific ceRNA networks in AAK patients lacks further study and the mechanisms by which they work are still unknown.

In this study, we constructed ceRNA networks to thoroughly understand their pathogenesis. Subsequently, we explored the immune microenvironment of AAK. Finally, we screened hub genes for predicting AAK occurrence based on the interaction of miRNAs and lncRNAs. We believe that this study will shed light on the pathogenesis of AAK, and provide potential biomarkers and new insights into its treatment.

## Materials and methods

### Patients and samples

Raw gene expression data and clinical information on GSE137996 and GSE137995 were downloaded from GEO dataset (http://www.ncbi.nlm.nih.gov/geo/). The GSE137996 dataset contains lncRNA and mRNA of 20 AAK patients and 20 healthy individuals, and the GSE137995 dataset contains miRNA data from 40 samples. Samples were taken from bulbar conjunctival cells, miRNA, lncRNA, and mRNA were detected, and all clinical information was available.

### Screening differentially expressed lncRNAs(DElncRNA), miRNAs (DEmiRNA), and mRNAs (DEmRNA)

Using the R package (limma), the expression profiles of 20 patients and 20 normal samples were compared to identify DEmRNAs, DElncRNAs, and DEmiRNAs. Genes were retained under the rule of a |log2 (fold-change) | > 1 and an adjusted *p* < 0.05.

### Weighted gene co-expression network analysis (WGCNA) for mRNA

A gene co-expression network analysis was specifically performed using mRNA data of the 40 microarray-measured samples from GSE137996 using the R package WGCNA (Langfelder and Horvath, 2008). A hierarchical clustering analysis of AAK and normal samples was performed, based on the expression of AAK to remove outlier samples. An adjacency matrix was transformed from the correlation matrix using the adjacency function (ai, j = | Cor (Xi, Xj)|β). The fit soft threshold power (β) was screened to ensure the construction of scale-free networks, based on Pearson’s correlation coefficient between two groups. Topology overlap measurement and robust network measurement were calculated in pairs based on the adjacency matrix. The best soft threshold was selected to construct a scale-free network. Then, the dissimilarity based on topological overlap was used as the input for unsupervised hierarchical clustering using the dynamic tree cutting algorithm (Langfelder et al., 2008). As a result of the TOM-based dissimilarity measure, average linkage hierarchical clustering was implemented, and genes with similar expression modes were classified into the same modules by step-by-step network construction and module detection with the following parameters: the softPower = 4, minModuleSize = 30, and mergeCutHeight = 0.35. The module eigengenes (MEs) represents the first principal component-related module, which is considered to represent all genes in the module. Eigengenes were performed to identify modules that are significantly associated with a disease. The whole process of WGCNA was performed using the R program (Li et al., 2018).

### Functional enrichment analysis

The module genes obtained by WGCNA and the differential expressed genes obtained by limma analysis were intersected to obtain crossed differentially expressed mRNAs (co-DEmRNAs). Gene Ontology (GO) biological functions and Kyoto Encyclopedia of Genes and Genomes (KEGG) pathway enrichment of co-DEmRNAs were analyzed by R packages “DOSE,” “clusterProfiler”, and“pathview” and visualized by the “enrichplot” package. The significant enrichment threshold was set as *p*-value < 0.05. Transcription factors (TFs) of DEmiRNA and GO annotation were realized by Funrich software (version 3.1.3) (Pathan et al., 2015), which revealed the TFs enrichment analysis of DEmiRNAs and biological processes (BP), cellular components (CC), and molecular functions (MF) of the miRNAs separately.

### Construction of lncRNA-miRNA-mRNA related ceRNA network

A miRcode database (http://www.mircode.org/download.php) was used to integrate evidence for direct interaction between DElncRNA and DEmiRNA (Jeggari et al., 2012). MultiMiR packages (Ru et al., 2014) were used to predict validated DEmiRNA-DEmRNAs pairs based on fourteen databases. Finally, ceRNA networks based on differentially expressed genes were constructed and visualized using Cytoscape software (version 3.9.1).

### Analyses of the ceRNA network-related hub genes

GO annotation and visualization of the hub genes were performed by the Metascape database (https://metascape.org/gp/index.html#/main). Next, the abundance of infiltrating immune cells of 40 samples was estimated and analyzed by the CIBERSORTx algorithm (http://cibersortx.stanford.edu/), based on running with batch correction and 100 permutations. Single-sample gene set enrichment analysis (ssGSEA) was performed to evaluate the correlation of immune function between AAK patients and the control group.

### Validation and clinical characters of hub genes

The top 10 hub genes from ceRNA network were screened in the PPI network using the MCC algorithm based on the CytoHubba plugin without checking the first‐stage nodes (Chin et al., 2014). Then ROC analyses of hub genes were performed using the R package pROC and genes with AUC greater than 0.9 were selected (Robin et al., 2011). Next, the samples were divided into the high-risk group and the low-risk group according to the median value of the screened hub genes, and GSEA was used to compare the differences in signaling pathways between the two groups and explore possible molecular mechanisms. Finally, we determined the expression levels of the hub genes with gender and stage of AAK and screened out genes that related to clinical characters.

### Statistics analysis

R software (R version 4.1.3) was used for all statistical analyses, and the “ggplot2” and “pheatmap” packages were used for graphical visualization. Statistical significance was defined as *p* < 0.05, and all *p*-values were two-tailed. The predictive accuracy of the disease prognostic model was assessed by performing a ROC curve analysis. The Mann-Whitney test was used to compare the proportion of tumor-infiltrating immune cells.

## Results

### Identification of differently expressed genes

**FIGURE 1:**
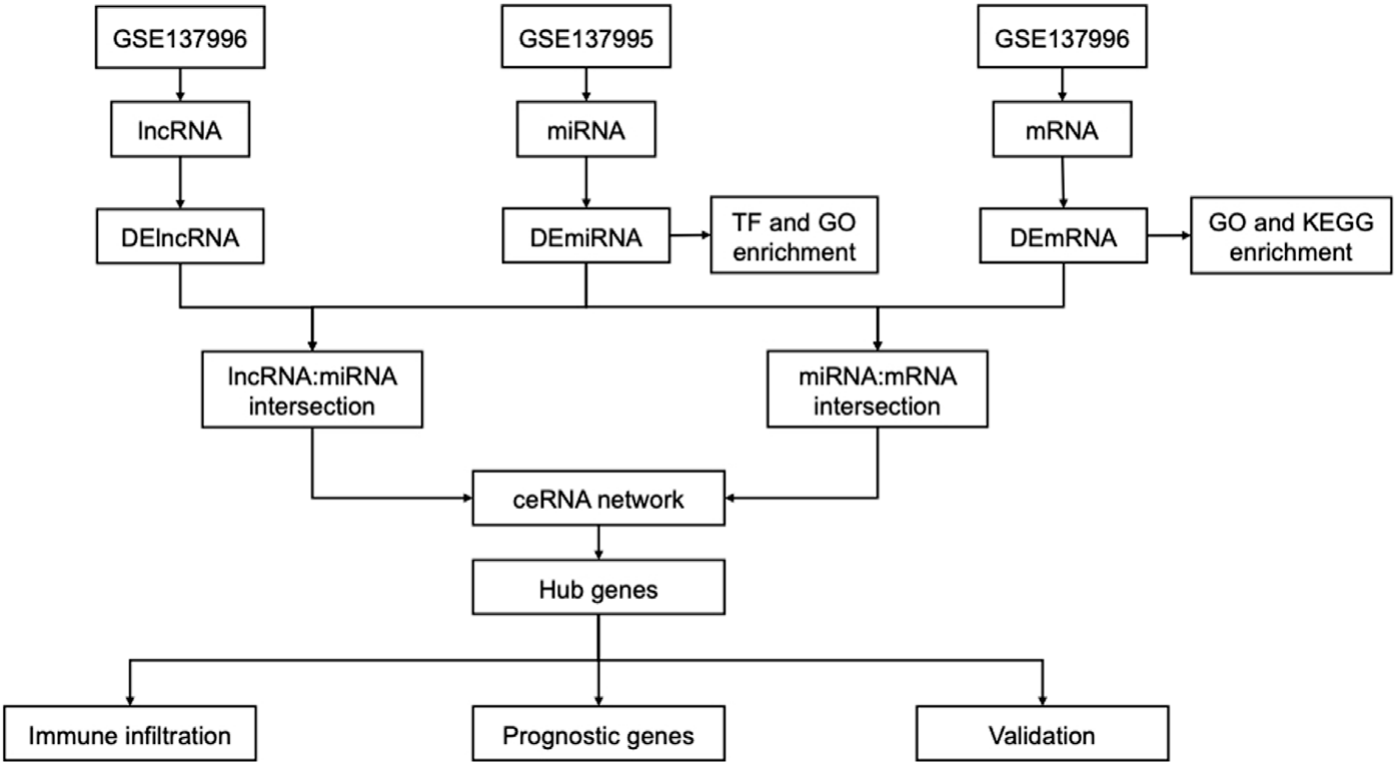
Flow chart of identification of ceRNA-related network hub genes in AAK.

**FIGURE 2:**
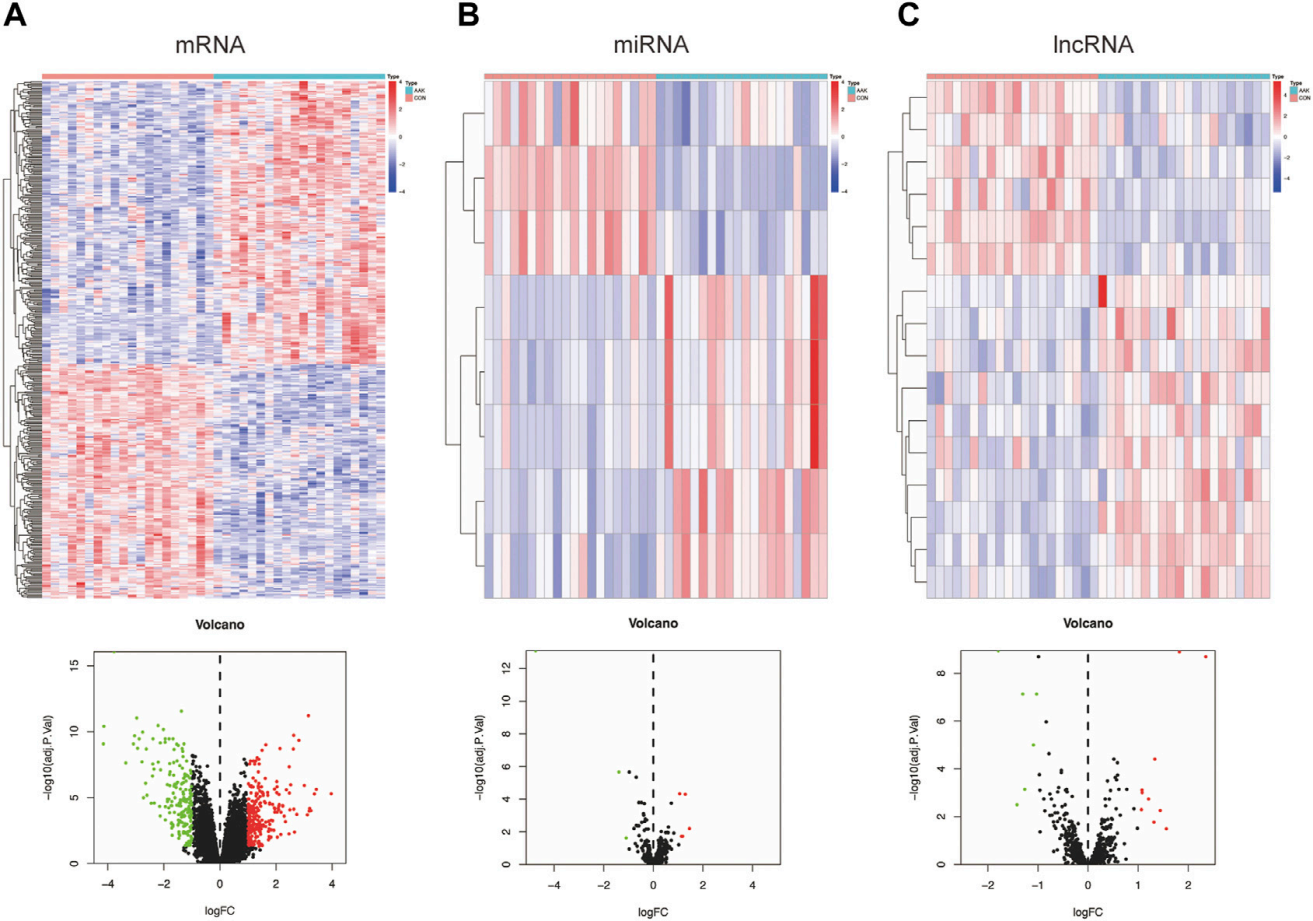
Differentially expressed genes between the AAK group and the control group. **(A)** Heatmap and volcano plot of differentially expressed mRNAs between AAK and control group. **(B)** Heatmap and volcano plot of differentially expressed miRNAs between AAK and control group. **(C)** Heatmap and volcano plot of differentially expressed lncRNAs between AAK and control group. Red points represent up-regulated genes. Green points represent down-regulated genes. Black points represent genes with no significant difference; AAK: aniridia-associated keratopathy.

The flow chart is shown in Figure 1. Based on annotation files downloaded from the GENCODE database, the expression profile of GSE13996 was divided into mRNA files containing 17,696 genes that can encode proteins and files that contain 1,981 lncRNAs. Meanwhile, we filtered 2449 miRNAs from GSE13995. Differential expression profiling was performed on the three RNAs, respectively. 422 differential expressed mRNAs (DEmRNAs), including 230 up-regulated genes and 192 down-regulated genes, were found in the mRNA expression profile, shown in the heatmap and a volcano plot (Figure 2A); 8 differential expressed miRNA (DEmiRNAs), including 5 up-regulated and 3 down-regulated were found in the miRNA expression profile (Figure 2B); 16 differential expressed lncRNA (DElncRNAs), including 10 up-regulated and 6 down-regulated were found in the lncRNA expression profile (Figure 2C). Supplementary Table S1 showed the detailed differently expressed genes**.**

### AAK-related WGCNA modules and genes

**FIGURE 3:**
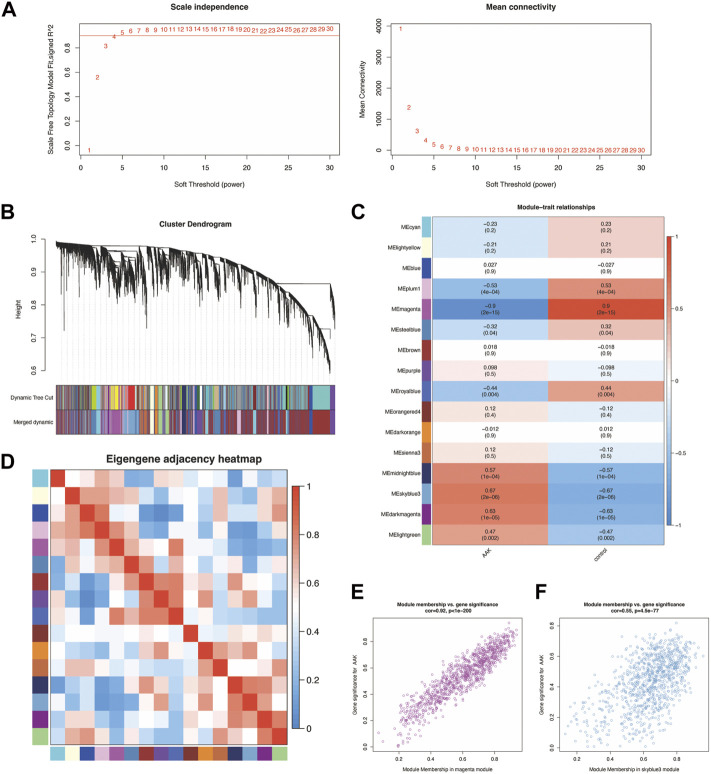
Construction of weighted co-expression network and module analysis. **(A)** With 0.90 being decided as scale independence, the power value of 4 was selected as the soft threshold of the adjacency matrix; **(B)** The branches of the cluster dendro-gram correspond to the 16 gene modules, each piece of the leaves on the cluster dendrogram corresponding to a different gene module. **(C)** The magenta module (r = −0.9, *p* = 2 × 10^−15^) module and the skyblue3 (r = 0.67, *p* = 2 × 10^−6^) module were the most strongly correlated with AAK. **(D)** Heat map of the eigengene adjacency. The color bars on the left and below indicate the modules for each row or column; **(E)** Correlation between module membership of magenta module and gene significance with AAK (cor = 0.92, *p* = 10^−200^). **(F)** Correlation between module membership of skyblue3 module and gene significance was shown in cor = 0.55 and *p* = −4.5 × 10^−77^.

To identify groups of genes with highly similar binding “signatures,” we adapted WGCNA to describe the correlation patterns of AAK, as it was one of the best methods for the construction of large networks in an unsupervised manner. The WGCNA package in R was applied to construct a co-expression network using the expression values of mRNA included in the 40 samples from the GSE137996 dataset. No sample was excluded from subsequent analysis. The scale-free topology network model was built to study gene expression networks. Based on the correlation coefficients for genes in the cohort, the adjacency matrix was transformed from the correlation matrix, with its power value of 4 as the soft threshold (Figure 3A). The scale independence was 0.90, and the mean connectivity of the co-expressed network was solid enough, ensuring a scale-free network (Figure 3B). Sixteen non-overlapping modules were constructed, and two highly AAK-correlated modules were detected (Figures 3C,D). The magenta module (r = −0.9, *p* = 2 × 10–15) module and the skyblue3 (r = 0.67, *p* = 2 × 10–6) module were strongly correlated with AAK. We obtained 2001 genes through this step, 1040 from the magenta module and 961 from the skyblue3 one. Correlation between module membership of magenta module and gene significance with AAK was shown in cor = 0.92, *p* = 10^−200^ and skyblue3 module in cor = 0.55 and *p* = −4.5 × 10^−77^ (Figures 3E,F). In addition, we performed GO and KEGG enrichment on two modules separately. They are both enriched in the same biological process involved in ossification, fatty acid metabolic process, and cell−cell adhesion via plasma−membrane adhesion molecules, etc (Supplementary Figures S1A,B), and they were both enriched in the same pathway involved in PI3K−Akt signaling pathway, Neuroactive ligand−receptor interaction, Cytokine−cytokine receptor interaction, etc., (Supplementary Figures S1C,D).

### Gene functional enrichment based on co-DEmRNA and DEmiRNA

**FIGURE 4:**
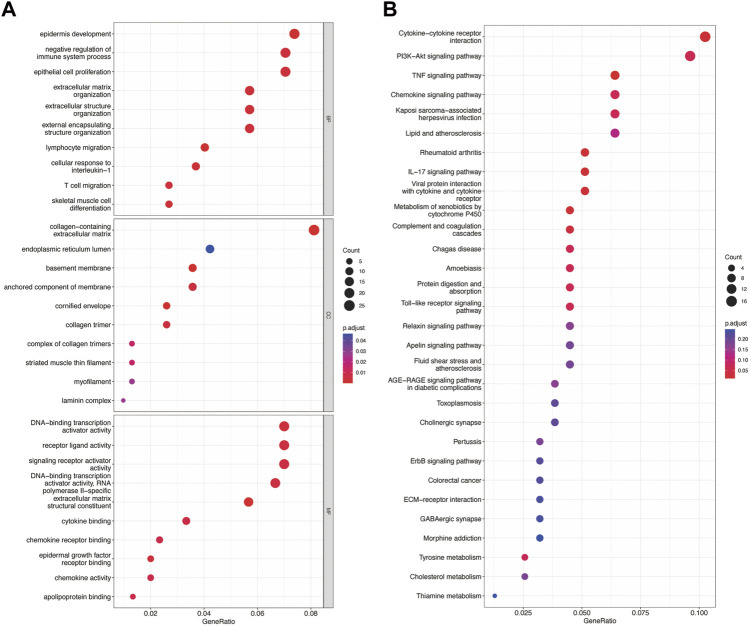
GO and KEGG enrichment of differential expression of mRNA. **(A)** The top 10 enrichment of BP, CC, and MF of differential expression of mRNA. **(B)** The top 30 enrichment of KEGG of differential expression of mRNA.

By intersecting the genes of the magenta and skyblue3 module and DEmRNA, we obtained 315 co-DEmRNA, including 157 upregulated and 158 downregulated. GO analysis was performed to search for biological functions (Figure 4A). We found that among the top ten enriched biological processes of co-DEmRNA, lymphocyte migration, cellular response to interleukin-1, and negative regulation of immune system process were all related to the function of immunity. Cellular component (CC) analysis showed co-DEmRNA were mainly enriched in the collagen−containing extracellular matrix, endoplasmic reticulum lumen, and basement membrane. Molecular function (MF) analysis showed co-DEmRNA mainly enriched in DNA−binding transcription activator activity, receptor ligand activity, and signaling receptor activator activity. These results indicated co-DEmRNA was involved in immunity. KEGG was also used to investigate the enriched pathways. The most enriched pathways were the cytokine-cytokine receptor interaction and the PI3K-Akt signaling pathway (Figure 4B), both of which were involved in immune processes (Giannone et al., 2020; Chauhan et al., 2021).

**FIGURE 5:**
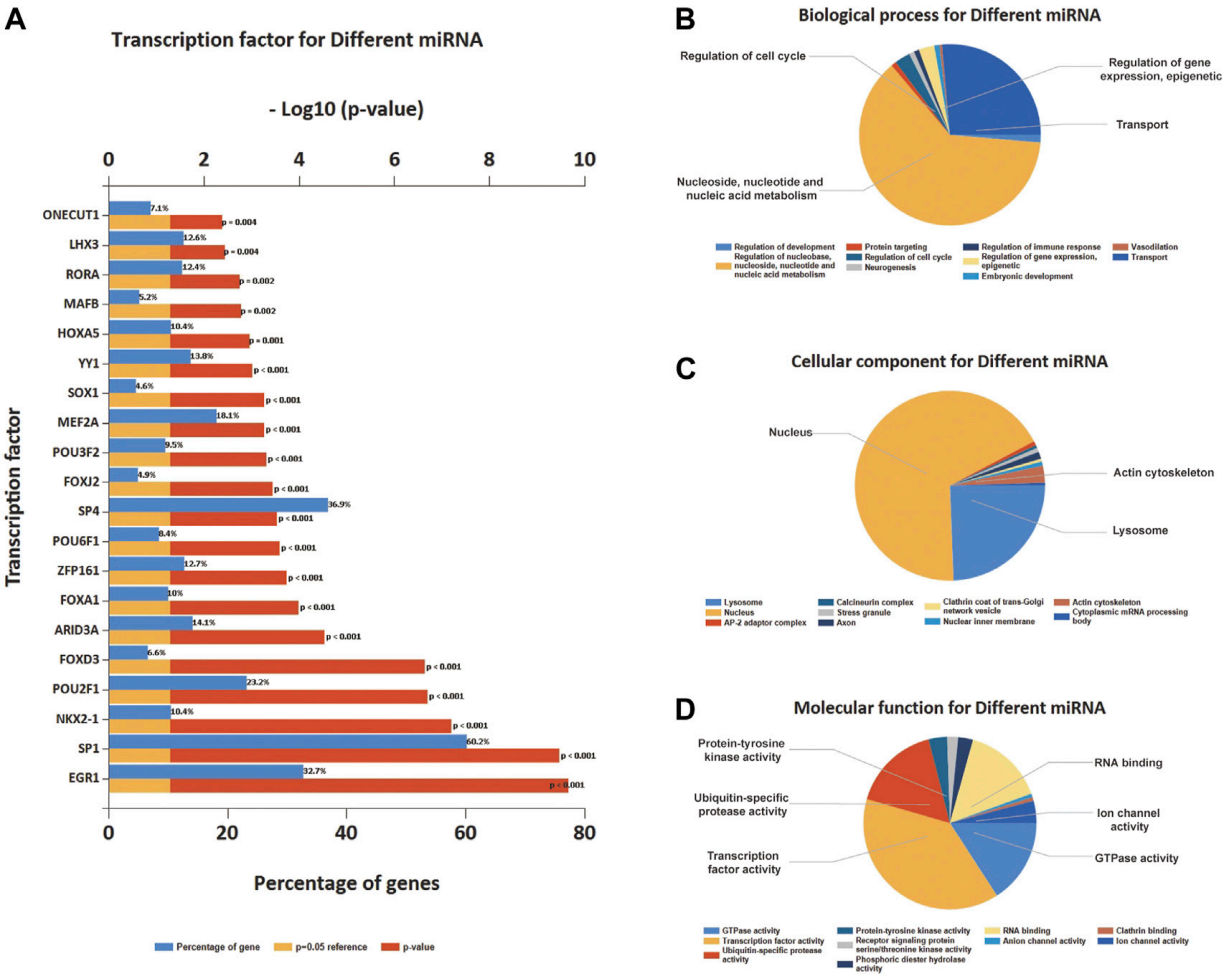
Transcription factors (TFs) enrichment and GO enrichment analysis of differential expression of miRNA. **(A)** The top 20 TFs were enriched based on differential expression of miRNA. **(B)** BP enrichment of the top 10 on differential expression of miRNA. **(C)** CC enrichment of the top 10 on differential expression of miRNA. **(D)** MF enrichment of the top 10 on differential expression of miRNA.

As for the DEmiRNA, the transcript factor (TF) enrichment analysis result is presented in Figure 5A, where the blue bar and the red bar illustrated the percentage of genes that miRNA enriched in TFs, and the *p*-value, respectively; The percentage of genes enriched for miRNA in TFs was shown. Enrichment analysis of the miRNAs based on GO revealed the ten most significant functional enrichments in BP, CC, and MF. Specifically, genes were mostly enriched in the regulation of nucleobase, nucleoside, nucleotide, and nucleic acid metabolism on BP and mostly distributed in the nucleus and nuclear inner membrane part related to CC (Figures 5B,C); as for MF, genes were particularly enriched in transcription factor activity, transcription regulator activity, and transporter activity, DNA binding, and ubiquitin-specific protease activity (Figure 5D).

### Construction of CeRNA Network in AAK

**FIGURE 6:**
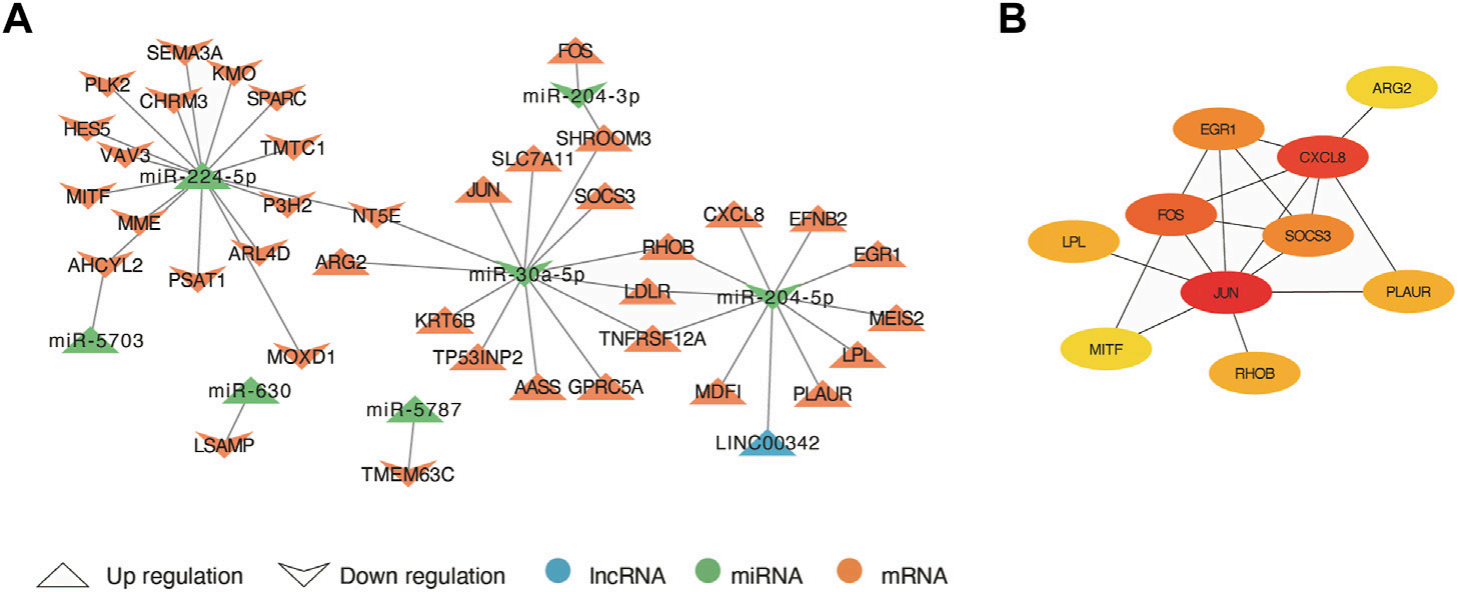
The lncRNA-miRNA-mRNA competing endogenous RNA (ceRNA) network and hub genes. **(A)** The arrow up triangle indicated upregulation genes in AAK patients; The arrow down triangle indicated downregulation genes in AAK patients; The blue represented lncRNAs; The green represented miRNAs; The orange represented mRNAs. **(B)** The hub genes were selected from the ceRNA network using the MCC algorithm.

Based on the ceRNA theory, lncRNAs compete for the binding of miRNA response elements (MREs) to manipulate the activity of mRNA. Thus, we constructed a ceRNA network of lncRNA-miRNA-mRNA using DElncRNA, DEmiRNA and co-DEmRNA to further elucidate the interaction among the three and visualized with Cytoscape (Figure 6A). There were 38 ceRNA-related genes in the network, including 20 up-regulated genes and 18 down-regulated genes excluding genes without DElncRNA linkages. Up-regulated LINC00342 can down-regulate miR-204-5p. In addition, miR-5787, miR-5703, miR-630, and miR-224-5p were upregulated and has-miR-204-3p and has-miR-30a-5p downregulated in the network. They may regulate those 38 genes and their regulatory network was of interest. Finally, using the MCC algorithm in the cytoHubba plugin, we further obtained ten hub genes namely *JUN, CXCL8, FOS, SOCS3, EGR1, RHOB, PLAUR, LPL, MITF*, and *ARG2* (Figure 6B).

### Immune infiltration analysis of AAK based on ceRNA-related genes

**FIGURE 7:**
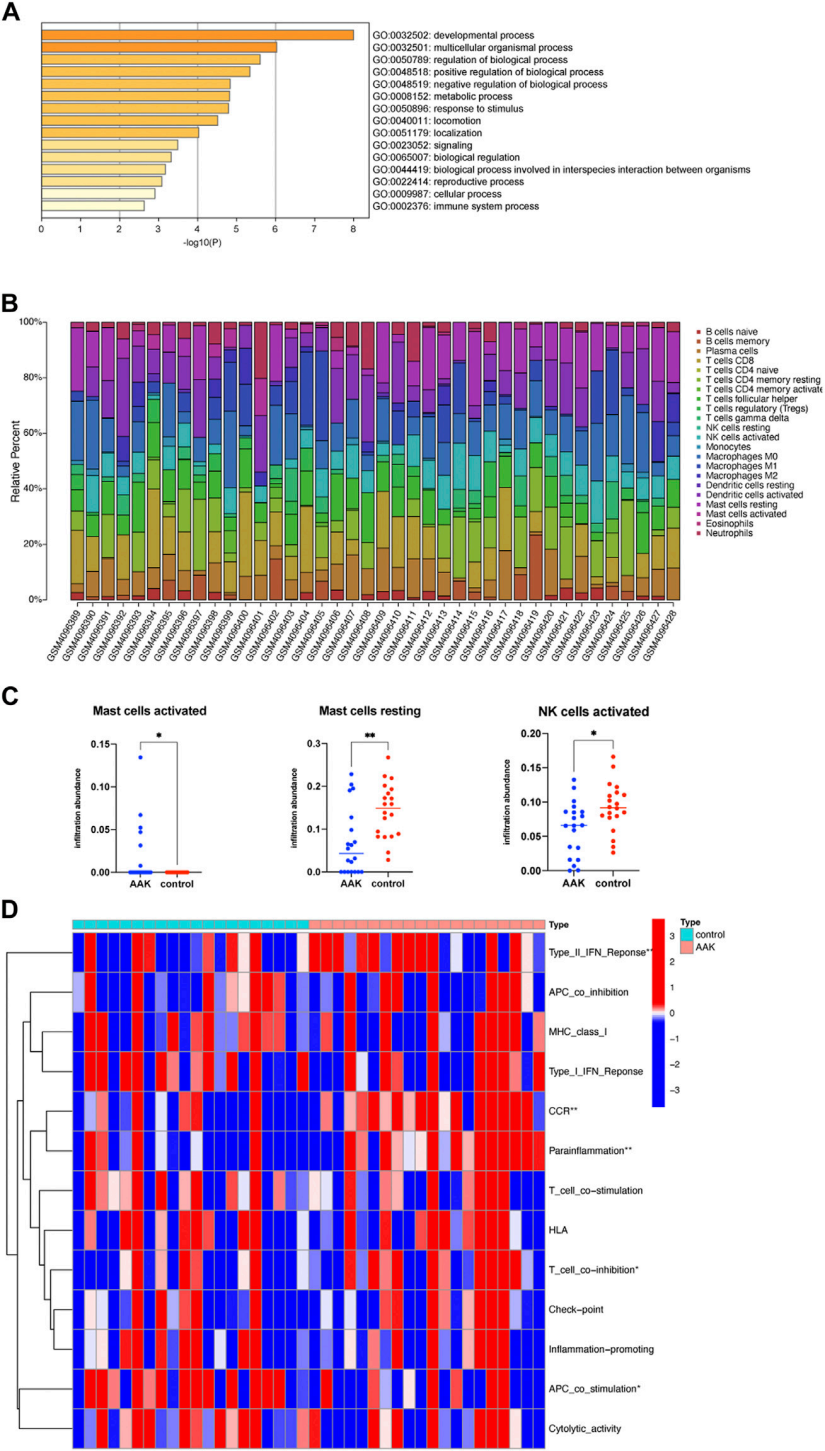
Immune function analysis. **(A)** GO enrichment of ceRNA-related genes, involving the immune system process. **(B)** CIBERSORTX immune cell infiltrates analysis. The percentage of 22 types of immune cells in each sample. **(C)** Activated Mast cells were more but resting mast cells and activated NK cells were less in the AAK group. **(D)** Further analysis of immune function based on the ssGSEA indicated that Type II INF Response, CCR, parainflammation, T cell co-stimulation, and APC co-stimulation were different between the two groups. *p < 0.05, **p < 0.01; ***p < 0.001.

We further performed GO enrichment analysis on the 38 related genes obtained from the ceRNA network. We enriched 15 biological processes with GO analysis, and we consider the developmental process and the immune system process to deserve further attention (Figure 7A). Combined with the enrichment results of co-DEmRNA, which was involved in the developmental process and immunity, we speculated that immune infiltration may participate in the occurrence of AAK. Thus, we calculated the ratio of 22 immune cells using the CIBERSORTX algorithm and plotted it in Figure 7B. Comparing the immune cells of the two groups, we found that infiltrating fraction of activated Mast cells increased in the AAK group while resting Mast cells decreased; the infiltrating fraction of activated NK cells decreased in the AAK group (Figure 7C). We then used ssGSEA to score immune-related functions and further analyzed differences in immune responses between the two groups. AAK scored higher in Type II INF Response, CCR, Parainflammation, and T cell co-stimulation, while APC co-stimulation showed the opposite trend (Figure 7D).

### Verification of hub genes and clinical significance

**FIGURE 8:**
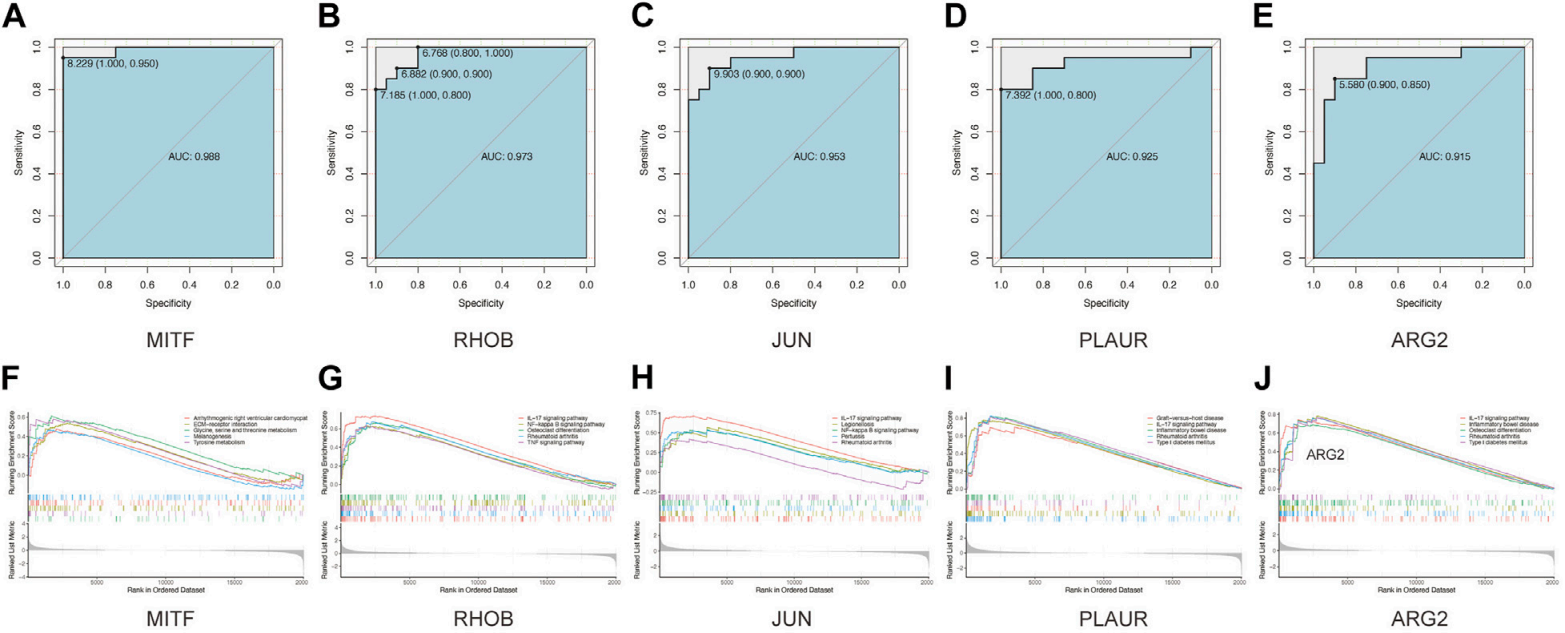
GSEA analyses and ROC curves of hub genes. **(A–E)**. The performance of using hub genes to predict AAK: MITF-AUC:0.988; RHOB-AUC:0.973; JUN-AUC:0.953; PLAUR-AUC:0.925; ARG2-AUC:0.915. **(F–J)** GAES analysis showed that Arrhythmogenic right ventricular cardiomyopathy, ECM−receptor interaction, Glycine, serine and threonine metabolism Melanogenesis, and Tyrosine metabolism. FOSB enriched in IL−17 signaling pathway Legionellosis, Osteoclast differentiation, Rheumatoid arthritis, and TNF signaling pathway. JUN enriched in IL−17 signaling pathway, Legionellosis, NF−kappa B signaling pathway, Pertussis, and Rheumatoid arthritis. PLAUR enriched in Graft−versus−host disease, IL−17 signaling pathway, Inflammatory bowel disease, Rheumatoid arthritis, and Type I diabetes mellitus. ARG2 enriched in IL−17 signaling pathway, Inflammatory bowel disease, Osteoclast differentiation, Rheumatoid arthritis, and Type I diabetes mellitus.

**FIGURE 9:**
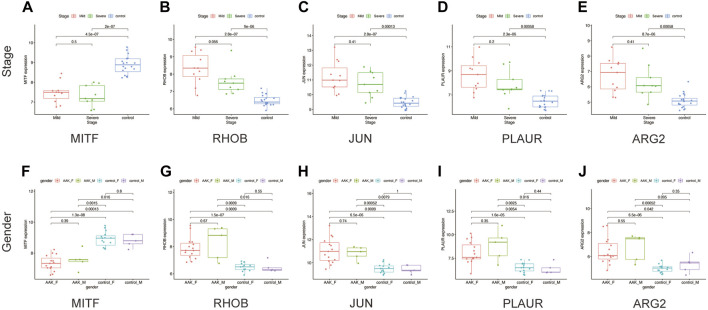
The clinical characteristics of hub genes. **(A–E)** The expression of five hub genes in different stages. **(F–J)** The expression of five hub genes in males and females between AAK and control patients.

Next, we seek to explore whether the ten hub genes (Figure 6B) can be used to predict the occurrence of AAK as independent genes. By applying logistics regression analysis between diagnosis and gene expression (Supplementary Table S2), we drew the receiver operating characteristic curve (ROC) of these hub genes and calculated the area under the curve (AUC). Genes with AUC>0.9 were considered to have satisfying predictive power, which were *MITF* (AUC = 0.988); *RHOB*(AUC = 0.973), *JUN*(AUC = 0.953), and *PLAUR* (AUC = 0.925), *ARG2* (AUC = 0.915) (Figure 8A–E). Meanwhile, we investigated specific signaling pathways related to these five hub genes and explored the potential molecular mechanism of AAK progression based on ssGSEA. Enrichment of the top five pathways for these five genes was shown (Figures 8F–J). Except for *MITF*, the top five enriched pathways of the other four genes all contained IL-17 signaling pathway, indicating that IL-17 signaling pathway may play an important role in the occurrence and development of AAK. We then analyzed the expression levels of the five hub genes. All genes did not differ by gender. *RHOB, JUN, PLAUR,* and *ARG2* elevated in AAK patients, while *MITF* was down-regulated (Figures 9A–E). Notably, although there was no statistical difference in gene expression between the mild group and the severe group, *RHOB, JUN, PLAUR,* and *ARG2* showed higher expression during the mild stage, suggesting that they may become effective biomarkers for early diagnosis (Figures 9F–J).

## Discussion

A ceRNA regulatory network was constructed for AAK patients with limbal stem cell deficiency. MiR-30a-5p, miR-204-5p, and miR-224-5p have connections to most of the genes in the network. Previous research revealed that miR-204-5p, an inhibitor of corneal neovascularization, was downregulated in severely vascularized corneas (Latta et al., 2020). Our results demonstrated that the upregulation of LINC00342, which was associated with macrophage M1 (Li et al., 2022) can down-regulate the expression of miR-204-5p, and subsequently up-regulate *CXCL8, EFNB2, EGR1, MEIS2, LPL, PLAUR, MDFI, TNFRSF12A, LDLR,* and *RHOB* genes. There are also studies showing that miR-30a-5p can regulate the endothelial to mesenchymal transition, which was a key link between inflammation and vascular calcification (Ciavarella et al., 2021). In uveal melanoma, miR-224-5p expressed lower compared to normal tissue and was involved in the proliferation, invasion, and migration via regulating the expression of *PIK3R3* and *AKT3* (Li et al., 2019). In conclusion, this network revealed the mechanism by which differently expressed genes are regulated at the transcriptome level.

The progression of AAK may be associated with immune cell infiltration. Studies have shown that inflammation can modulate limbal stem cell function and may lead to limbal stem cell deficiency in some cases (Puangsricharern and Tseng, 1995; Li et al., 2007). In this study, co-DEmRNA was considered to regulate immune system process, epithelial cell proliferation, and extracellular structure organization. The ceRNA-related genes were also enriched in immune system process. Among the symptoms of AAK are erosion of the corneal surface, epithelial thinning or loss, inflammation, vascularization, and chronic progressive opacification (Latta et al., 2021). The density of mature dendritic cells is significantly elevated in aniridia individuals compared to normal individuals (Lagali et al., 2020). Likewise, our immune infiltration analysis found that activated Mast cells were elevated considerably while resting mast cells decreased in AAK patients, and activated NK cells decreased in patients with AAK, indicating that these two types of immune cells may be involved in the progression of AAK. IL-17 is a pro-inflammatory cytokine and is released predominantly by activated Th17 cells, invariant natural killer T (NKT) cells, and mast cells (Cua and Tato, 2010). Many eye diseases, such as uveitis, dry eye, and keratitis, involve IL-17 (Qin et al., 2019; Zhong et al., 2021; Wang et al., 2022). Of note, in addition to down-regulated *MITF*, the GSEA analysis of the other four up-regulated hub genes and the KEGG analysis of co-DEmRNA also enriched the IL-17 signaling pathway, which indicates that IL-17 played an important role in the pathogenesis of AAK.

*MITF* is a microphthalmia-associated transcription factor and its germline mutations are associated with clinically distinct disorders (Ma et al., 2019). A previous study showed that both *PAX6* and *MITF* are required for pigment epithelium development *in vivo* (Bharti et al., 2012). Similarly, in AAK patients with *PAX6* mutations, we observed that *MITF* was also downregulated. When used as a gene for predicting the occurrence of AAK, its AUC also reached 0.988.

*RHOB* is a key regulator of multiple cellular processes and can be rapidly induced by a variety of stimuli to regulate cell proliferation, survival, and apoptosis. A recent study has shown that hypoxia significantly upregulates the expression of *RHOB* (Huang et al., 2017). Concurrent studies suggested that *RHOB* was genetically required for pathogenic retinal angiogenesis (Almonte-Baldonado et al., 2019). Likewise, *RHOB* was also up-regulated in AAK patients. *PLAUR* encodes the receptor for urokinase plasminogen activator and could be related to tumor growth and angiogenesis (Lakka et al., 2003). *ARG2* is one of two isoforms of arginase. *ARG2* impaired endothelial autophagy through the regulation of mTOR and PRKAA/AMPK signaling (Xiong et al., 2014) and global deletion of *ARG2* limited I/R-induced retinal layer disruption, fundus abnormalities, and albumin extravasation by altering mitochondrial dynamics and function (Shosha et al., 2021). Consistent with these studies, we observed that *RHOB, PLAUR,* and *ARG2* were upregulated in both mild and severe stages, indicating the upregulation of these genes may play an important role in the occurrence of AAK.

Currently, AAK patients were treated symptomatically by either eye drops (artificial tears, serum eye drops) or surgical treatment (amniotic membrane transplantation, corneal transplants, etc.). (Landsend et al., 2021) In a previous study, aloe emodin inhibited colon cancer cell migration/angiogenesis by downregulating MMP-2/9, RhoB, and VEGF (Suboj et al., 2012). Masatoshi Hara etc. considered that an ARG2-specific inhibitor may effectively treat kidney ischemia-reperfusion injury (Hara et al., 2020). Hence, the five hub genes may provide a reference for the drug development of AAK.

However, there were certain limitations in this study. Additional *in vitro* and *in vivo* experiments, such as cell culture and establishment of animal models are required to further investigate the potential mechanisms underlying AAK. The present study may provide a research basis for the diagnosis and treatment of AAK.

## Conclusion

We constructed a ceRNA network, revealed that AAK was associated with immune infiltration, and identified hub genes with high confidence (AUC>0.9) that can be used for analysis and diagnosis. We hope our results may provide a reference value for future researchers.

## Data Availability

Publicly available datasets were analyzed in this study. The names of the repository/repositories and accession number(s) can be found in the article/Supplementary Material.

## References

[B1] Almonte-Baldonado R. Bravo-Nuevo A. Gerald D. Benjamin L. E. Prendergast G. C. Laury-Kleintop L. D. (2019). RhoB antibody alters retinal vascularization in models of murine retinopathy. J. Cell. Biochem. 120 (6), 9381–9391. 10.1002/jcb.28213 30536763

[B2] Bharti K. Gasper M. Ou J. Brucato M. Clore-Gronenborn K. Pickel J. (2012). A regulatory loop involving PAX6, MITF, and WNT signaling controls retinal pigment epithelium development. PLoS Genet. 8 (7), e1002757. 10.1371/journal.pgen.1002757 22792072 PMC3390378

[B3] Chauhan P. Nair A. Patidar A. Dandapat J. Sarkar A. Saha B. (2021). A primer on cytokines. Cytokine 145, 155458. 10.1016/j.cyto.2021.155458 33581983

[B4] Chin C. H. Chen S. H. Wu H. H. Ho C. W. Ko M. T. Lin C. Y. (2014). cytoHubba: identifying hub objects and sub-networks from complex interactome. BMC Syst. Biol. 8 (4), S11. 10.1186/1752-0509-8-S4-S11 25521941 PMC4290687

[B5] Ciavarella C. Motta I. Vasuri F. Fittipaldi S. Valente S. Pollutri D. (2021). Involvement of miR-30a-5p and miR-30d in endothelial to mesenchymal transition and early osteogenic commitment under inflammatory stress in HUVEC. Biomolecules 11 (2), 226. 10.3390/biom11020226 33562690 PMC7915105

[B6] Cua D. J. Tato C. M. (2010). Innate IL-17-producing cells: The sentinels of the immune system. Nat. Rev. Immunol. 10 (7), 479–489. 10.1038/nri2800 20559326

[B7] Giannone G. Ghisoni E. Genta S. Scotto G. Tuninetti V. Turinetto M. (2020). Immuno-metabolism and microenvironment in cancer: Key players for immunotherapy. Int. J. Mol. Sci. 21 (12), E4414. 10.3390/ijms21124414 32575899 PMC7352562

[B8] Hara M. Torisu K. Tomita K. Kawai Y. Tsuruya K. Nakano T. (2020). Arginase 2 is a mediator of ischemia-reperfusion injury in the kidney through regulation of nitrosative stress. Kidney Int. 98 (3), 673–685. 10.1016/j.kint.2020.03.032 32739205

[B9] Huang G. Su J. Zhang M. Jin Y. Wang Y. Zhou P. (2017). RhoB regulates the function of macrophages in the hypoxia-induced inflammatory response. Cell. Mol. Immunol. 14 (3), 265–275. 10.1038/cmi.2015.78 26388235 PMC5360878

[B10] Jeggari A. Marks D. S. Larsson E. (2012). miRcode: a map of putative microRNA target sites in the long non-coding transcriptome. Bioinformatics 28 (15), 2062–2063. 10.1093/bioinformatics/bts344 22718787 PMC3400968

[B11] Käsmann-Kellner B. Seitz B. (2014). [Aniridia syndrome: Clinical findings, problematic courses and suggestions for optimization of care ("aniridia guide")]. Ophthalmologe. 111 (12), 1145–1156. 10.1007/s00347-014-3060-x 25475188

[B12] Lagali N. Wowra B. Dobrowolski D. Utheim T. P. Fagerholm P. Wylegala E. (2018). Stage-related central corneal epithelial transformation in congenital aniridia-associated keratopathy. Ocul. Surf. 16 (1), 163–172. 10.1016/j.jtos.2017.11.003 29133179

[B13] Lagali N. Wowra B. Fries F. N. Latta L. Moslemani K. Utheim T. P. (2020). Early phenotypic features of aniridia-associated keratopathy and association with PAX6 coding mutations. Ocul. Surf. 18 (1), 130–140. 10.1016/j.jtos.2019.11.002 31734509

[B14] Lakka S. S. Gondi C. S. Yanamandra N. Dinh D. H. Olivero W. C. Gujrati M. (2003). Synergistic down-regulation of urokinase plasminogen activator receptor and matrix metalloproteinase-9 in SNB19 glioblastoma cells efficiently inhibits glioma cell invasion, angiogenesis, and tumor growth. Cancer Res. 63 (10), 2454–2461. 12750266

[B15] Landsend E. C. S. Lagali N. Utheim T. P. (2021). Congenital aniridia - a comprehensive review of clinical features and therapeutic approaches. Surv. Ophthalmol. 66 (6), 1031–1050. 10.1016/j.survophthal.2021.02.011 33675823

[B16] Langfelder P. Horvath S. (2008). Wgcna: an R package for weighted correlation network analysis. BMC Bioinforma. 9, 559. 10.1186/1471-2105-9-559 PMC2631488 19114008

[B17] Langfelder P. Zhang B. Horvath S. (2008). Defining clusters from a hierarchical cluster tree: The dynamic tree cut package for R. Bioinformatics 24 (5), 719–720. 10.1093/bioinformatics/btm563 18024473

[B18] Latta L. Figueiredo F. C. Ashery-Padan R. Collinson J. M. Daniels J. Ferrari S. (2021). Pathophysiology of aniridia-associated keratopathy: Developmental aspects and unanswered questions. Ocul. Surf. 22, 245–266. 10.1016/j.jtos.2021.09.001 34520870

[B19] Latta L. Ludwig N. Krammes L. Stachon T. Fries F. N. Mukwaya A. (2020). Abnormal neovascular and proliferative conjunctival phenotype in limbal stem cell deficiency is associated with altered microRNA and gene expression modulated by PAX6 mutational status in congenital aniridia. Ocul. Surf. 19, 115–127. 10.1016/j.jtos.2020.04.014 32422284

[B20] Lee H. J. Colby K. A. (2013). A review of the clinical and genetic aspects of aniridia. Semin. Ophthalmol. 28 (5-6), 306–312. 10.3109/08820538.2013.825293 24138039

[B21] Li C. Su F. Liang Z. Zhang L. Liu F. Fan W. (2022). Macrophage M1 regulatory diabetic nephropathy is mediated by m6A methylation modification of lncRNA expression. Mol. Immunol. 144, 16–25. 10.1016/j.molimm.2022.02.008 35168108

[B22] Li J. Liu X. Li C. Wang W. (2019). miR-224-5p inhibits proliferation, migration, and invasion by targeting PIK3R3/AKT3 in uveal melanoma. J. Cell. Biochem. 120 (8), 12412–12421. 10.1002/jcb.28507 30825222

[B23] Li J. Zhou D. Qiu W. Shi Y. Yang J. J. Chen S. (2018). Application of weighted gene Co-expression network analysis for data from paired design. Sci. Rep. 8 (1), 622. 10.1038/s41598-017-18705-z 29330528 PMC5766625

[B24] Li W. Hayashida Y. Chen Y. T. Tseng S. C. (2007). Niche regulation of corneal epithelial stem cells at the limbus. Cell Res. 17 (1), 26–36. 10.1038/sj.cr.7310137 17211449 PMC3190132

[B25] Lim H. T. Kim D. H. Kim H. (2017). PAX6 aniridia syndrome: Clinics, genetics, and therapeutics. Curr. Opin. Ophthalmol. 28 (5), 436–447. 10.1097/ICU.0000000000000405 28598868

[B26] Lim H. T. Seo E. J. Kim G. H. Ahn H. Lee H. J. Shin K. H. (2012). Comparison between aniridia with and without PAX6 mutations: Clinical and molecular analysis in 14 Korean patients with aniridia. Ophthalmology 119 (6), 1258–1264. 10.1016/j.ophtha.2011.12.010 22361317

[B27] Ma X. Li H. Chen Y. Yang J. Chen H. Arnheiter H. (2019). The transcription factor MITF in RPE function and dysfunction. Prog. Retin. Eye Res. 73, 100766. 10.1016/j.preteyeres.2019.06.002 31242455

[B28] Pathan M. Keerthikumar S. Ang C. S. Gangoda L. Quek C. Y. Williamson N. A. (2015). FunRich: An open access standalone functional enrichment and interaction network analysis tool. Proteomics 15 (15), 2597–2601. 10.1002/pmic.201400515 25921073

[B29] Puangsricharern V. Tseng S. C. G. (1995). Cytologic evidence of corneal diseases with limbal stem cell deficiency. Ophthalmology 102 (10), 1476–1485. 10.1016/s0161-6420(95)30842-1 9097795

[B30] Qin X. H. Ma X. Fang S. F. Zhang Z. Z. Lu J. M. (2019). IL-17 produced by Th17 cells alleviates the severity of fungal keratitis by suppressing CX43 expression in corneal peripheral vascular endothelial cells. Cell Cycle 18 (3), 274–287. 10.1080/15384101.2018.1556059 30661459 PMC6380429

[B31] Robin X. Turck N. Hainard A. Tiberti N. Lisacek F. Sanchez J. C. (2011). pROC: an open-source package for R and S+ to analyze and compare ROC curves. BMC Bioinforma. 12, 77. 10.1186/1471-2105-12-77 PMC3068975 21414208

[B32] Ru Y. Kechris K. J. Tabakoff B. Hoffman P. Radcliffe R. A. Bowler R. (2014). The multiMiR R package and database: Integration of microRNA-target interactions along with their disease and drug associations. Nucleic Acids Res. 42 (17), e133. 10.1093/nar/gku631 25063298 PMC4176155

[B33] Salmena L. Poliseno L. Tay Y. Kats L. Pandolfi P. P. (2011). A ceRNA hypothesis: The rosetta stone of a hidden RNA language? Cell 146 (3), 353–358. 10.1016/j.cell.2011.07.014 21802130 PMC3235919

[B34] Shosha E. Fouda A. Y. Lemtalsi T. Haigh S. Fulton D. Ibrahim A. (2021). Endothelial arginase 2 mediates retinal ischemia/reperfusion injury by inducing mitochondrial dysfunction. Mol. Metab. 53, 101273. 10.1016/j.molmet.2021.101273 34139341 PMC8274341

[B35] Suboj P. Babykutty S. Valiyaparambil Gopi D. R. Nair R. S. Srinivas P. Gopala S. (2012). Aloe emodin inhibits colon cancer cell migration/angiogenesis by downregulating MMP-2/9, RhoB and VEGF via reduced DNA binding activity of NF-κB. Eur. J. Pharm. Sci. 45 (5), 581–591. 10.1016/j.ejps.2011.12.012 22227305

[B36] Wang J. Gong J. Yang Q. Wang L. Jian Y. Wang P. (2022). Interleukin-17 receptor E and C-C motif chemokine receptor 10 identify heterogeneous T helper 17 subsets in a mouse dry eye disease model. Am. J. Pathol. 192 (2), 332–343. 10.1016/j.ajpath.2021.10.021 35144761

[B37] Wang L. Yu T. Zhang X. Cai X. Sun H. (2021). Network integration analysis and immune infiltration analysis reveal potential biomarkers for primary open-angle glaucoma. Front. Cell Dev. Biol. 9, 793638. 10.3389/fcell.2021.793638 34926471 PMC8678480

[B38] Xiong Y. Yepuri G. Forbiteh M. Yu Y. Montani J. P. Yang Z. (2014). ARG2 impairs endothelial autophagy through regulation of MTOR and PRKAA/AMPK signaling in advanced atherosclerosis. Autophagy 10 (12), 2223–2238. 10.4161/15548627.2014.981789 25484082 PMC4502672

[B39] Ye Z. Li Z. He S. (2017). Long non-coding RNA associated-competing endogenous RNAs are induced by clusterin in retinal pigment epithelial cells. Mol. Med. Rep. 16 (6), 8399–8405. 10.3892/mmr.2017.7606 28944909

[B40] Zhang L. Dong Y. Wang Y. Gao J. Lv J. Sun J. (2019). Long non-coding RNAs in ocular diseases: New and potential therapeutic targets. Febs J. 286 (12), 2261–2272. 10.1111/febs.14827 30927500

[B41] Zhong Z. Su G. Kijlstra A. Yang P. (2021). Activation of the interleukin-23/interleukin-17 signalling pathway in autoinflammatory and autoimmune uveitis. Prog. Retin. Eye Res. 80, 100866. 10.1016/j.preteyeres.2020.100866 32422390

