# Supplementary figures and images for: Construction of ceRNA network and identification of hub genes in aniridia-associated keratopathy using bioinformatics analysis
Source: Front Genet. 2022 Sep 23;13:997581. doi: 10.3389/fgene.2022.997581 (PMC9537812; doi:10.3389/fgene.2022.997581)

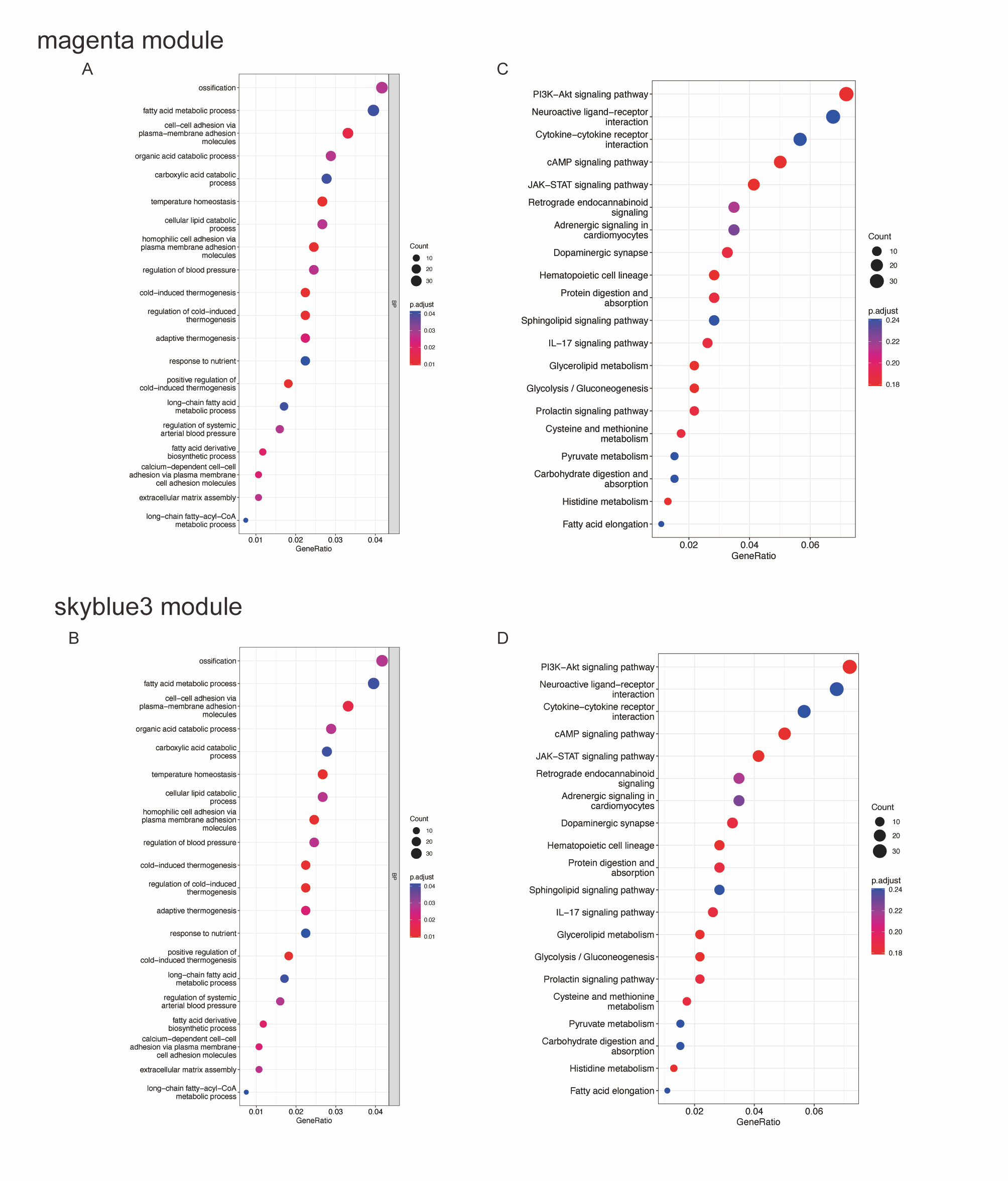

Supplement: Supplementary file 1 [file Image1.JPEG]
